# Childhood stunting and subsequent educational outcomes: a marginal structural model analysis from a South African longitudinal study

**DOI:** 10.1017/S1368980022001823

**Published:** 2022-11

**Authors:** Lateef B Amusa, Annah Vimbai Bengesai, Hafiz TA Khan

**Affiliations:** 1 Department of Statistics, University of Ilorin, Ilorin, Nigeria; 2 College of Law and Management Studies, University of KwaZulu-Natal, 4000 Durban, South Africa; 3 College of Nursing, Midwifery and Healthcare, University of West London, Brentford, UK

**Keywords:** Stunting, Marginal structural model, Confounding, South Africa

## Abstract

**Objective:**

To examine the association between childhood stunting and grade completion (as educational outcome) in South Africa.

**Design:**

Longitudinal study. Data were obtained using the National Income Dynamics Study over five waves (2008 to 2017). Children were tracked at wave 1 in 2008 until wave 5 in 2017 to determine their total years of schooling. We controlled for time-variant and time-varying confounding with a marginal structural model to estimate the associations between childhood stunting and subsequent grade completion.

**Setting:**

Nationally representative study of South African households.

**Participants:**

A total of 2629 children aged 2 and 3 years in 2008.

**Results:**

We observed a substantial decrease in the prevalence of stunting between wave 1 (28·2 %) and wave 4 (8·6 %). Our marginal structural model results suggest that childhood stunting was significantly associated with decreased odds (22 % less likely) of grade completion (OR = 0·78; 95 % CI: 0·40, 0·86; *P* = 0·015), while those who were only stunted during early childhood had a 29 % reduction in the odds of grade completion (OR = 0·71; 95 % CI: 0·51, 0·82; *P* = 0·020).

**Conclusion:**

These findings underscore the fact that stunting is a significant predictor of academic achievement, whose effects might be long-lasting.

For many years, poor academic achievement has been a significant area of concern in many countries^(1)^, prompting research that seeks to identify its correlates. Several factors that can lead to underperformance have been identified, including schooling quality, poverty, illness, age at enrolment, absenteeism and nutrition^(2,3)^. However, for young children, nutritional deficiencies are among the significant factors affecting academic achievement^(1)^. One possible manifestation of malnutrition is stunting (or low height-for-age), which some scholars argue is the best indicator of chronic undernutrition and a more accurate reflection of socio-economic inequalities^(4,5)^.

The prevalence of stunting in Africa is approximately 29 %, 8 % higher than the global average of 21 %^(5,6)^. The Eastern and Southern Africa regions account for the increasing share of the global total within the continent, with the rural population being the most vulnerable due to insufficient dietary intakes. Although South Africa has undergone a nutrition transition characterised by the prevalence of obesity and non-communicable disease similar to most developed countries^(7)^, stunting remains prevalent. The 2020 nutrition status report indicated that over 1·5 million (three out of ten) South African children are stunted^(8)^, surpassing countries like Gabon, Libya and Egypt, which have similar Human Development Indices to South Africa. These high stunting rates have also persisted, and efforts to rectify the situation through school-based nutrition programmes have proven ineffective^(7)^. This is not surprising given that by the time children reach school-going age, the effects of stunting are unlikely to be reversible.

Certainly, stunting is a significant public health issue associated with increased morbidity, unhealthy development and mortality from infections^(5)^. For instance, greater risk of metabolic syndrome has been found among adults who were stunted in early childhood^(9,10)^. This is because malnutrition in the early years can trigger ‘permanent epigenetic changes in metabolism’^(11)^, which, although necessary for survival, can result in rapid weight gain and obesity, increased risk hypertension and high blood sugar. For South Africa, this association cannot be ignored, as it poses serious public health consequences in a country already grappling with the burden of HIV and AIDS, among other diseases^(12)^. Apart from the increased morbidity, it is believed that poor linear growth observed in the first 24 months of life is associated with delayed cognitive development and poor academic achievement through a shared set of determinants (such as poor nutrition, repeated infection and inadequate psychosocial stimulation)^(13)^, leading to adverse outcomes affecting the entire lifecycle^(14)^, and even persisting into the labour force^(15)^. For instance, using the Young Lives (YL) from India, Ethiopia, Peru and Vietnam, and a multivariable probit regression approach, Crookston et al.^(16)^ found an inverse relationship between stunting at the age of 1 year and achievement in mathematics, reading comprehension, and receptive vocabulary. Casale^(17)^, who used data from four waves of the National Income Dynamics Study (NIDS, South Africa), found that children who had a low height-for-age in the early period were likely to complete fewer years of schooling by the age of 14 years than their peers who did not experience stunting. The South African Birth to Twenty Cohort Study also suggests that not just the quantity and quality of nutrition make a difference, but also the timing^(18,19)^. Even if their nutritional status improves, children who have been stunted in early childhood never quite catch up in academic outcomes^(20)^. In this way, malnutrition can hinder children from reaching their full potential. Although some studies have shown that this association is counteracted when controlling for psychosocial and environmental factors^(13)^, for a country like South Africa with one of the most unequal education systems globally^(21)^, stunting adds an additional layer that cannot be ignored.

Undoubtedly, the adverse effects of stunting in early childhood are well documented. However, some studies have questioned the stability of stunting over time, arguing that recovery is possible beyond 2 years and in early adolescence^(22)^. Proponents of this view have used different approaches to support the claim of stunting recovery. For instance, Faye et al.^(23)^, who used the height-for-age Z-score (HAZ) slope modelling to estimate changes in linear growth, found that the incidence of recovery from stunting was approximately 45 %. In Casale’s study^(4)^, a sample aged 24–36 months at baseline was used, given that the prevalence of stunting often increases between birth and 2 years, peaking somewhere between the ages of 2 and 3 years. Thus, using a starting point for stunting too early can lead to misreporting of catch-up growth. However, this perspective, referred to in the literature as *catch-up growth* or *recovery from stunting,* has become contentious in nutrition research. For instance, Leroy et al.^(24)^ questioned the validity of this claim, arguing that both the methods and the measures often used are not adequate to estimate stunting recovery. Further, they recommend that catch-up growth can only happen under certain conditions, such as when growth-inhibiting conditions are controlled.

Considering these debates and adopting a time-varying confounding approach, this study utilised data from a population-based longitudinal study to measure the time-dependent confounding in the association between childhood stunting and academic achievement. We compared standard longitudinal methods with the marginal structural modelling approach of estimating causal effects using the inverse probability of treatment (IPT) weighting^(25)^. Marginal structural models (MSM) provide a framework for estimating time-varying exposures while controlling for both time-invariant and time-varying confounders that may be affected by previous exposures.

Given that stunting is an outcome of many factors, most of which are associated with unfavourable socio-economic and household circumstances^(26)^, we also included some of these and examined their interplay with stunting and educational attainment. Risk factors that have been identified in many studies include limited access to nutritional food, comorbidity of stunting and other illnesses^(27)^, parental education, family income, among others^(28)^. For instance, low-income families might have difficulties obtaining food, resulting in nutritional deficiencies in young children. In some cases, linear growth can falter due to other illnesses, and this co-occurrence can lead to profound negative development outcomes^(29)^. Several studies have also demonstrated the effect of parental education, particularly maternal education, on children’s outcomes, with maternal education considered to have far-reaching effects than any other determinant^(30)^. This is because educated parents can access health information, including following recommended feeding schemes. Although these correlates have been observed in many studies, their effect is likely to vary in space and time. Hence, there is a need for context-specific studies to provide deeper insight into their interplay with stunting and educational attainment.

## Methods

### Data

We used data from the five waves (2008–2017) of the NIDS. The NIDS, a nationally representative sample of the South African population, is a biennial longitudinal survey of the dynamic structure of households and changes in their health and well-being^(31)^. The NIDS uses a stratified, two-stage cluster sample design. Stratification was done at the district council level, while clustering was employed using the primary sampling unit. The first wave of the NIDS, which was conducted in 2008, comprised 26 776 successfully interviewed persons (95 % response rate) from 7296 responding households (69 % response rate). The same people were followed and re-interviewed, with new household members being included in the subsequent waves. The overall attrition rate between waves 1 and 2, waves 2 and 3, waves 3 and 4 was 21·95 %, 15·82 %, and 13·75 %, respectively^(32)^. Further details regarding the NIDS study design can be found in a published report^(33)^.

This study examines the impact of childhood stunting of children on subsequent educational outcomes, measured by grade completion. We thus followed a cohort of children aged at most 3 years in wave 1 (2008) until wave 5 (2017), when the youngest cohort members were at least aged 9 years and the oldest aged 12 years. This resulted in an eligible sample of 2629 children. Across all five waves of data collection, 830 children were not successfully interviewed or lost to follow-up in at least one of the waves (wave 1: *n* 67; wave 2: *n* 454; wave 3: *n* 374; wave 4: *n* 366; and wave 5: *n* 438). Further, we dropped those who were not successfully interviewed in any of the five waves (*n* 11), reducing the sample size to 2618.

### Measures

The NIDS data contain a wide range of information on, but not limited to, children’s nutritional, anthropometric and educational outcomes. Respondents provided self-reported and measured data both at baseline and follow-up.

### Outcome variable

In this study, grade completion was the outcome of interest. We used the available information on the highest grade completed by wave 5. By South African educational standards, a child must start Grade 1 in the year they turn 7. Thus, when observed in wave 5, all the children in our sample should be enrolled in school, with the youngest cohort (aged 9 years) having completed Grade 3 (and enrolled in Grade 4) if (i) they were on time and (ii) had progressed one grade per year. Thus, we quantified for analysis with a binary indicator of whether or not a child completed Grade 3 or higher in 2017 (wave 5).

### Exposure variable

In our analyses, we defined the exposure variable using a binary indicator of stunting of a given child for a particular time point in the study period. We measured childhood stunting in this study by the z-score of the child’s HAZ. HAZ and other anthropometric indicators were calculated using the WHO international child growth standards and references for children aged under 5 years and adolescents^(34,35)^. Based on these growth standards, we defined a stunted child as having a HAZ less than two standard deviations (HAZ < –2) below the median value of a healthy reference population. To avoid inflated z-scores, the NIDS z-scores used months as the unit of analysis. This is in contrast to the *zanthro macro scores* where the default unit of analysis is the year and a 2-year-old is considered to be 2 years 0 months old. Unfortunately, this leads to children being compared to a reference population that might be up to 364 d younger than they are. Rather, in the NIDS, children aged 30 months (2 years and 6 months), for instance, were considered to be 2-year-old^(32)^. The NIDS data were also pre-cleaned, and biologically implausible values were coded as missing in line with WHO guidelines^(4)^.

### Confounders

**Fig. 1:**
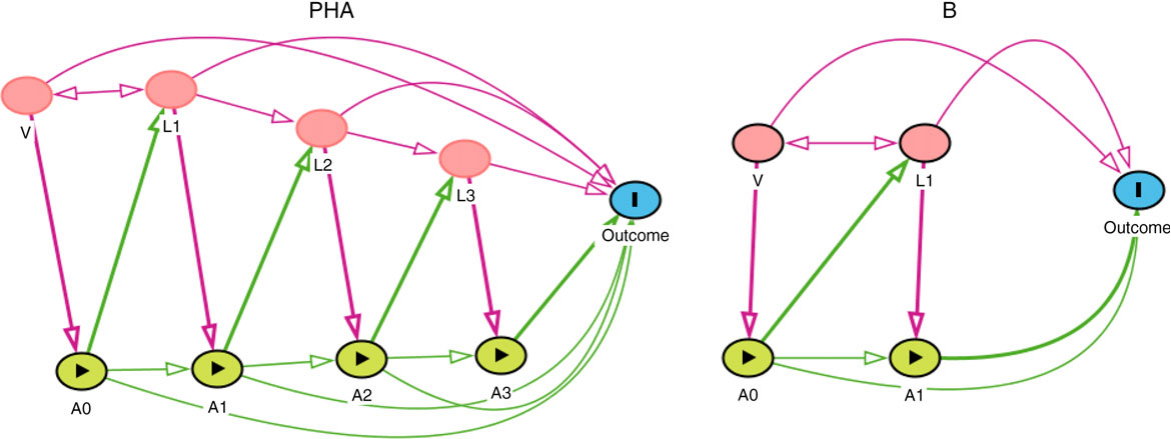
Simplified directed acyclic graphs of the hypothetical causal relationship between time-dependent stunting on subsequent educational attainment. Numeric subscripts 0, 1, 2 and 3 denote the baseline period (wave 1), 1st, 2^nd^ and 3rd follow-ups, respectively. V represent the time-invariant baseline covariates (gender, race, birth weight and parental education); A represents stunting status; L represents the time-varying covariates (household receipt of government grants, child lived with both parents, child has any illness or disability, type of residential area, household income and household size) at time points other than the baseline. Panel A: stunting effect was captured only for the early childhood years (within the first 5 years); Panel B: stunting effect was captured till the later childhood years (as late as the age of 9 years)

We controlled for possible confounders for the relationship between stunting status and grade completion outcome. The confounders were chosen based on theory and previous research findings, and their potential relationships were depicted in a causal diagram (see Fig. 1). Time-invariant covariates in the analysis include gender, race (African and others) and the child’s birth weight (kg). We further included the highest school grade completed by the parents. Mother and father’s education were combined into a composite variable, parental education, based on who completed a higher number of years of education.

**Fig. 2:**
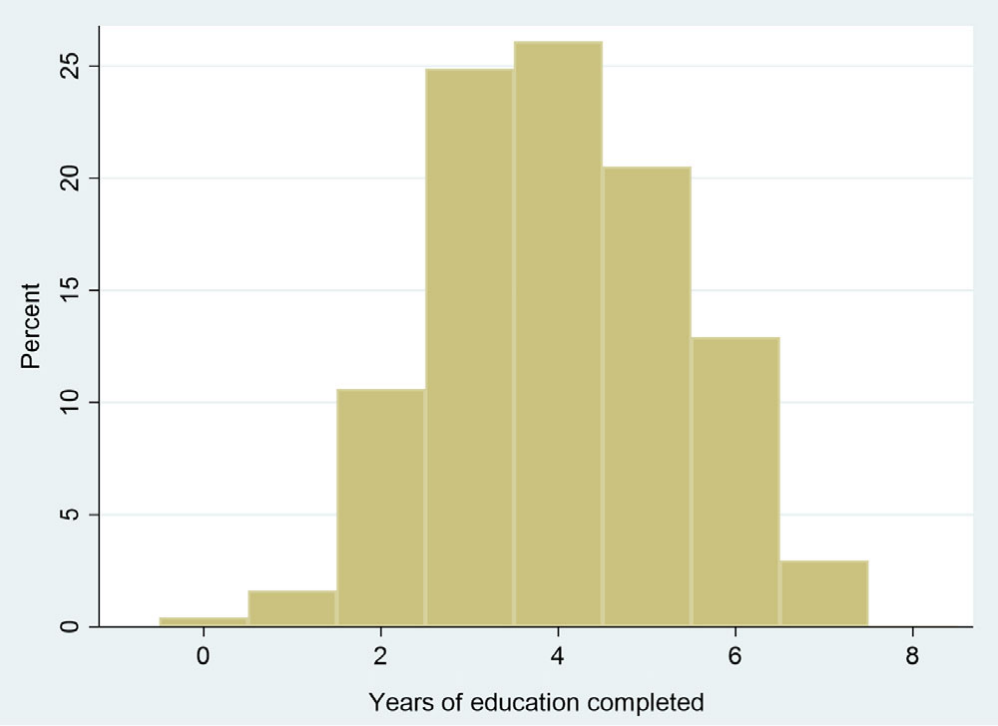
Educational attainment of cohort members in 2017 (aged 9–12 years)

The following variables were measured throughout the study and included as time-varying covariates: an indicator for whether both parents live with the child, the number of household residents, an indicator for whether a child has/had any serious illnesses or disabilities, type of residential area (rural and urban), natural log of per capita household income (Rands) and an indicator for whether the household receives a child support grant.

### Statistical analysis

Marginal structural models are a suitable statistical approach for longitudinal data to control the confounding effect of time-dependent confounders on time-varying treatments or exposures. Instead of applying IPT weights to each subject, MSM build upon the widely used propensity score weighting technique in a two-stage process. In the first stage, IPT weights are estimated for every subject-time point in the study period. These weights create a pseudo-population where treatment is unconfounded by subject-specific characteristics^(36)^. In the second stage, a weighted pooled regression model including only the exposure history can estimate the causal effect of the exposure of interest on the study outcome. MSM lead to unbiased estimates under the following assumptions: exchangeability (no unmeasured confounders); consistency (no ambiguity in defining exposure); positivity (nonzero probability of each possible exposure value given all combinations of covariates) and correct model specification^(37)^.

Since children stunting status is a time-varying variable, we adopted inverse probability weighted estimation of MSM to provide an unbiased causal effect estimate of childhood stunting on grade completion. Further, as shown in Fig. 1, MSM appropriately adjusts the confounding effects that vary over time from one wave to another, including some of these confounders with both a confounding and mediating role in the causal pathway linking the exposure and the outcome.

We applied the stabilised version of the IPT weights (SW) to reduce variability and produce more efficient estimates^(25)^ as follows:

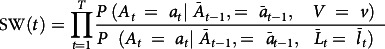





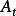
 denotes the time-varying exposure at time *t*, 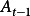 denotes the exposure history before time *t*, while 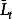 are the time-dependent covariates through time *t*, and *V* represents the vector of baseline covariates.

To estimate the denominator of the stabilised weights, we fitted a binary logistic regression of stunting status at time *t* ( 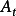 ) on previous times stunting status ( 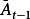 ), 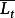 and 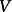 . We similarly estimated the numerator, apart from excluding the time-varying covariates. To assess violations of the positivity and model specification assumptions, we verified that the mean of the stabilised inverse probability weights was close to 1·0, with no extreme weights. To further confirm non-violation of positivity, we checked that no particular subgroups in the sample were rarely or never in the exposed group.

We fitted two separate models based on the corresponding hypothetical scenarios and causal pathways depicted in Fig. 1(a) and (b), respectively. We assumed in the first scenario that the stunting impact was only observed in the early years (within the first 5 years), while in the second scenario, we assumed that the stunting effect was captured till the later years (as late as the age of 9 years). Our MSM were logit models, which allow the estimation of OR. We fitted weighted (using the IPT weights) logistic regression models to estimate the probability of grade completion, comparing stunted and non-stunted children. Standard errors were cluster-robust to account for clustering observations within subjects over time^(38)^. For comparison, we estimated the conventional unweighted regression model (both unadjusted and adjusted) to assess the magnitude of time-dependent confounding.

### Missing value analysis

There was at least one missing value for some variables, including whole wave missingness. We assumed that the data were missing at random and adopted multiple imputation via chained equations^(39)^ to allow that the missingness mechanisms might depend on measured covariates. We performed five imputations and applied Rubin’s rule^(40)^ to get combined estimators for the risk ratios, including CI from the imputed data. In a sensitivity analysis, we repeated the analyses of the fitted models with data on complete cases to evaluate how results compare with those from our main analyses (Appendix Table 1, Supplementary file).

Apart from multiple imputations, which were implemented in Stata due to ease of use, all the analyses were carried out in R version 4.0.2^(41)^.

## Results

The mean birth weight of the cohort members was 3·1 kg (±0·6 kg), most of them were African (83·8 %), and a little above half were males (50·5 %). Their parents’ number of years of education was 10·3 years on average (±2·8 years). At baseline, 28·2 % of the children were stunted, the prevalence of which decreased substantially over the three follow-ups, decreasing to 8·6 % in wave 4. Household income increased over time, with a mean log of income of 7·7 Rands at baseline increasing to 8·4 Rands at wave 4. The proportion of children whose parents lived with them and those who lived in the rural area decreased slightly over time (Table 1).

**Table 1:** Sample characteristics, NIDS waves 1–4

Time-invariant variables	Wave 1 (baseline)							
	*n*	%	*n*	%	*n*	%	*n*	%
Gender*								
Male	1323	50·5						
Female	1295	49·5						
Race*								
African	2195	83·8						
Others	423	16·2						
Birth weight (kg)^b^								
Mean	3·10							
sd	0·64							
Parental education (number of years of schooling)^b^								
Mean	10·29							
sd	2·77							

Time-varying variables	Wave 1 (baseline)		Wave 2		Wave 3		Wave 4	
Exposure: stunting status*								
Not stunted	1125	71·8	1005	76·9	1769	84·4	2014	91·4
Stunted	443	28·2	302	23·1	328	15·6	189	8·6
Household receipt of child support grant*								
No	590	22·6	492	22·4	475	21·0	435	19·2
Yes	2018	77·4	1701	77·6	1787	79·0	1834	80·8
Lived with both parents*								
No	1833	71·7	1607	74·2	1716	76·1	1758	78·9
Yes	725	28·3	559	25·8	538	23·9	476	21·3
Has any illness or disability*								
No	2425	95·9	2100	96·8	2169	96·2	2135	94·7
Yes	105	4·1	69	3·2	85	3·8	119	5·3
Residence*								
Rural	1523	58·2	1389	57·0	1368	55·2	1327	54·0
Urban	1095	41·8	1049	43·0	1108	44·8	1131	46·0
Log of household income (Rands)^b^								
Mean	7·70		7·86		8·14		8·42	
sd	0·95		0·95		0·90		0·87	
Household size^b^								
Mean	6·62		6·98		6·71		6·48	
sd	3·28		3·67		3·61		3·46	

NIDS, National Income Dynamics Study.

All non-missing data are presented.

* Values expressed as *n* (%).

b Values expressed as mean (sd).

At baseline, in comparison to children who were not stunted, children who had stunted growth were more likely to be males (*P* < 0·01), to have a smaller birth weight (*P* < 0·001) and belong to households with smaller income (*P* < 0·001) (Table 2). Figure 1 shows the distribution of educational attainment of the sample at wave 5. The mean years of education completed (range: 0–8) was 4·0 (± 1·4 years). Only a small proportion of the children (12·6 %) had not completed Grade 2 or higher in 2017.

**Table 2:** Distribution of baseline covariates according to stunting status

Covariates	Not stunted (*n* 945, 71·8 %)		Stunted (*n* 408, 28·3 %)	
	*n*	%	*n*	%
Gender†,**				
Male	549	48·8	250	56·4
Female	576	51·2	193	43·6
Race†				
African	975	86·7	376	84·9
Others	150	13·3	67	15·1
Birth weight (kg)‡,***				
Mean	3·16		2·92	
sd	0·59		0·58	
Parental education (number of years of schooling)‡				
Mean	10·40		10·03	
sd	2·77		2·83	
Household receipt of child support grant†				
No	217	19·3	81	18·3
Yes	906	80·7	361	81·7
Lived with both parents†				
No	843	74·9	320	72·2
Yes	282	25·1	123	27·8
Has any illness or disability†				
No	1063	95·9	420	95·2
Yes	45	4·1	21	4·8
Residence†				
Rural	715	63·6	277	62·5
Urban	410	36·4	166	37·5
Log of Household income (Rands)‡,***				
Mean	7·73		7·56	
sd	0·92		0·88	
Household size‡				
Mean	6·74		6·81	
sd	3·30		3·40	

All non-missing data are presented.

** *P*-value < 0·01.

*** P-value < 0·001

† Values expressed as *n* (%).

‡ Values expressed as mean (sd).

Table 3 shows the OR estimates for stunting status on the children’s educational attainment. In all the models fitted, we found that stunting, compared with not stunting, was associated with an overall reduction in the odds of grade completion (completing Grade 5 or higher in 2017). Our MSM indicated that comparing children who had stunted growth with children who were not stunted, there was a 22 % significant reduction in the odds of grade completion (OR = 0·78; 95 % CI: 0·40, 0·86). Similar MSM estimates were observed for stunting effects limited to early childhood stunting only (Model B). Stunting was associated with a 29 % significant reduction in the odds of grade completion (OR = 0·71; 95 % CI: 0·51, 0·82).

**Table 3:** Associations between stunting and subsequent educational attainment

		OR	95 % CI	*P*-value
Model A	MSM*	0·78	0·40, 0·86	0·015
	Conventional model (unadjusted)†	0·67	0·53, 0·85	0·002
	Conventional model (adjusted)‡	0·73	0·57, 0·94	0·017
Model B	MSM*	0·71	0·51, 0·82	0·020
	Conventional model (unadjusted)†	0·63	0·49, 0·80	< 0·001
	Conventional model (adjusted)‡	0·68	0·53, 0·88	0·004

Model A: controlled for stunting effect from early to late childhood.

Model B: controlled for stunting effect from early childhood only.

* Marginal structural model (MSM) estimated from pooled logistic regression model (inverse probability of treatment weighted), controlling for the baseline and time-varying covariates listed in Table 1.

† Shown for comparison purposes only. Unadjusted model included only the time-varying intercept and stunting status.

‡ Shown for comparison purposes only. Adjusted model used the same pooled logistic regression model used in the MSM but without inverse probability of treatment weighting.

Slightly weaker associations were observed for the unweighted pooled logistic regression model not adjusting for time-dependent confounding. The unadjusted OR of grade completion was 0·67 (95 % CI: 0·53, 0·85), and the addition of baseline covariates to the model decreased the OR to 0·73 (95 % CI: 0·57, 0·94), which still suggests a detrimental effect of stunting. Effect estimates were consistent with what was observed for stunting effects limited to early childhood stunting only (Model B): OR 0·63 (95 % CI: 0·49, 0·80) in the crude model and OR 0·68 (95 % CI: 0·53, 0·88) in the multivariable-adjusted model.

Our results for sensitivity analysis of missing values produced identical results to our main analyses across models (Appendix A). However, the effect estimates were more imprecise with relatively wider CI and insignificant estimates. For example, the MSM analysis for the association between stunting and grade completion produced the following estimates for both early and childhood stunting: OR = 0·79 (95 % CI: 0·50, 1·09) and early childhood stunting only: OR = 0·80 (95 % CI: 0·46, 1·31). Results from our conventional unweighted models were further consistent.

## Discussion

Using the NIDS data, this study examined stunting trajectories over time and academic achievement, measured by grade completion. To the best of our knowledge, this is the first study to utilise a marginal structural modelling approach, which controls time-dependent confounding, to examine the relationship between stunting and educational outcomes in South Africa. Although there were minor differences in our MSM estimates and the conventional longitudinal methods used in other studies, the former is recommended when time-dependent confounding is likely, given its theoretically solid appeal.

Using data from five waves of the NIDS study, we found that a substantial proportion of children in our sample were stunted at baseline, and this figure decreased over 6 years. These findings are in line with statistics from other South African studies, which have estimated stunting to be between 26 and 38 %^(18,19)^, including the 2016 South African Demographic and Health Survey, whose estimate for under-5 stunting was approximately 27 %^(42)^. In essence, the findings confirm that stunting in South Africa remains prevalent and continues to be an alarming burden^(7)^.

Regarding the change in stunting, similar trends have been observed in previous South African^(4,19)^ as well as global studies^(43,44)^. For example, using the National Health and Nutrition Examination Survey, Shisana, Labadarios^(19)^ found that stunting changes from 26 % for girls and 27 % for boys aged 0–3 years to approximately 10 % for girls and 14 % for boys aged 4–6 years. Using the NIDS data, Desmond and Casale^(18)^ also found evidence of changes in stunting, with over 80 % having recovered from stunting by 4–6 years. In Zhang’s^(44)^ study, the authors found that although there was some evidence of catch-up growth in HAZ, most of the children in their sample remained in the same HAZ categories. However, we take caution of the earlier arguments against interpreting this improvement as evidence of catch-up growth^(24)^. Instead, these changes indicate that the child’s HAZ is less of an outlier than when it was previously measured^(18)^. This potential improvement does not overlook the importance of the first 1000 d in child growth; instead, they have implications for interventions targeting the nutritional needs of older children such as the school nutrition programme in South Africa^(45)^.

Our consistent results across all the considered estimation techniques, including the MSM, reinforced our hypothesis that childhood malnutrition significantly and adversely affected children’s educational outcomes, measured by grade completion in this study. Results from our MSM mainly show that stunted children were 22 % less likely to have completed the number of grades expected for their age. A further restriction of stunting to early childhood (only the first five years) produced even a more substantial effect: a 29 % reduction in the odds of grade completion. These findings are comparable to a recent analysis of the NIDS data^(46)^, which noted lower educational attainment and the risk of grade repetition among stunted children.

There are several reasons why stunting is negatively associated with educational outcomes. Stunted children are more likely to enter school later than their peers due to a combination of nutritional deficits and delayed cognitive development^(13,47)^. Reporting on Tanzania, Alderman, Hoogeveen^(48)^ noted that stunted children are often considered not ready to start school at the minimum age. Apart from delayed enrolment, stunted children are also likely to repeat grades owing to poor academic performance or missing classes due to food shortages^(1,46)^. Stunting has also been associated with poorer psychosocial functioning, including being less happy, fussy and more apathetic^(49)^. These factors have also been inversely associated with educational attainment^(1,4)^. Thus, although stunting is not part of the ‘mechanistic path leading to poor cognitive development’,^(13)^ it still increases the educational disadvantage of stunted children from entry and reduce their likelihood of being in the correct grade for age throughout the schooling cycle.

These findings are informative for South Africa as they confirm that stunting fuels educational inequality. In a country with one of the highest educational inequalities globally, stunting adds another layer of inequality that cannot be easily ignored. Although strides have been made to improve childhood nutrition through the school nutrition programme, the COVID-19 pandemic has worsened the plight of undernourished children^(50)^. The intermittent closure of many schools, including ECDs as the country works towards containing the pandemic, have meant that millions of children who have been beneficiaries of school-based nutrition programmes have been moving in and out of food security during the pandemic^(44)^. Therefore, issues of undernutrition will remain relevant in South Africa for some time.

Although this study has many strengths, for example, the longitudinal nature of the data and the time-varying approach, it is not without limitations. First, given that HAZ is a relative measure of stunting, there is a possibility that this measure might overestimate any height improvements. Second, we acknowledge that other factors might be associated with both stunting and grade completion beyond what we included in our models. Thus, the possibility of omitted variable bias should be taken into consideration, making it challenging to investigate any casual links. As such, only associations were estimated.

Despite these limitations, our findings are still relevant in South Africa and the scholarship because they confirm prior studies that have shown that stunting is still prevalent in South Africa and a cause for concern. Moreover, our findings indicate that even with the possibility of stunting status varying with time, poor linear growth in the first 36 months is still negatively associated with educational outcomes. Therefore, greater attention must be placed on understanding the mechanisms which underlie linear growth failure as well as interventions that prevent its incidence from the onset. Interventions which also target individual and household-level characteristics that make children vulnerable to stunting and poor educational attainment are also needed.

## Supplementary material

For supplementary material accompanying this paper visit https://doi.org/10.1017/S1368980022001823. click here to view supplementary material
