# Supplementary material for: Childhood stunting and subsequent educational outcomes: a marginal structural model analysis from a South African longitudinal study
Source: Public Health Nutr. 2022 Aug 26;25(11):3016–24. doi: 10.1017/S1368980022001823 (PMC9991553; doi:10.1017/S1368980022001823)
Supplement: Supplementary file 1 [file S1368980022001823sup001.docx]

**Appendix Table 1:** Sensitivity Analysis: Associations between stunting and subsequent educational attainment using complete cases for missing values.

|  | |  | Odds Ratio | 95% CI | P-value |
| --- | --- | --- | --- | --- | --- |
| Model A | MSM * | | 0.67 | 0.52, 0.86 | 0.002 |
|  | Conventional model (unadjusted) Ɨ | | 0.77 | 0.56, 1.06 | 0.111 |
|  | Conventional model (adjusted) § | | 0.79 | 0.50, 1.09 | 0.23 |
| Model B | MSM* | | 0.41 | 0.34, 0.50 | <0.0001 |
|  | Conventional model (unadjusted) Ɨ | | 0.78 | 0.51, 1.22 | 0.279 |
|  | Conventional model (adjusted) § | | 0.80 | 0.46, 1.31 | 0.33 |

Model A: controlled for stunting effect from early to late childhood

Model B: controlled for stunting effect from early childhood only

* Marginal structural model estimated from pooled logistic regression model (inverse probability of treatment weighted), controlling for the baseline and time-varying covariates listed in Table 1.

Ɨ Shown for comparison purposes only. Unadjusted model included only the time-varying intercept and stunting status

§ Shown for comparison purposes only. Adjusted model used the same pooled logistic regression model used in the MSM but without inverse probability of treatment weighting.

**Appendix Table 2:** Pooled logistic regression: Associations between other covariates and subsequent educational attainment

|  | Model A | | | |  | Model B | | | |
| --- | --- | --- | --- | --- | --- | --- | --- | --- | --- |
|  | OR | 95% CI | | P-value |  | OR | 95% CI | | P-value |
| Wave | 0.937 | 0.904 | 0.972 | 0.000 |  | 0.958 | 0.929 | 0.987 | 0.005 |
| Parental education (number of years) | 1.061 | 1.011 | 1.113 | 0.019 |  | 1.069 | 1.022 | 1.117 | 0.005 |
| Lived with both parents | 0.899 | 0.689 | 1.173 | 0.430 |  | 0.784 | 0.524 | 1.174 | 0.220 |
| Has any illness or disability | 0.529 | 0.372 | 0.753 | 0.001 |  | 0.828 | 0.462 | 1.483 | 0.514 |
| Log of household income (Rands) | 1.168 | 1.018 | 1.339 | 0.028 |  | 1.089 | 0.946 | 1.252 | 0.228 |
| Household size | 0.979 | 0.946 | 1.013 | 0.224 |  | 0.984 | 0.945 | 1.026 | 0.448 |
| Type of residence (Urban) | 0.911 | 0.685 | 1.210 | 0.513 |  | 0.997 | 0.745 | 1.335 | 0.984 |
| Household receipt of child grant | 1.164 | 0.919 | 1.473 | 0.201 |  | 1.208 | 0.921 | 1.584 | 0.171 |
| Gender (female) | 0.509 | 0.380 | 0.683 | 0.000 |  | 0.503 | 0.372 | 0.679 | 0.000 |
| Race (African) | 1.260 | 0.843 | 1.881 | 0.254 |  | 1.157 | 0.804 | 1.665 | 0.432 |
| Birth weight | 1.129 | 0.923 | 1.381 | 0.236 |  | 1.115 | 0.921 | 1.350 | 0.262 |

Model A: controlled for stunting effect from early to late childhood

Model B: controlled for stunting effect from early childhood only

**Appendix Figure 1:** STATA output of Multiple imputation via chained equations.

Note: Variables with suffixes 1, 2, 3, 4 correspond to waves 1, 2, 3, 4, respectively.

Abbreviations: bweight, birth weight; hhincome, household income; hhsize, household size; paedu, parental education; y1, educational outcome; grant, household receipt of child support grant; stunt, indicator for stunting status; healthy, has any illness or disability; parentres, lived with both parents; residence, type of place of residence.
